# Interaction of Human Lymphocyte Scavenger Receptors CD5 and CD6 with Toxins from *Naja haje*, *Androctonus australis* and *Apis mellifera* Venoms

**DOI:** 10.3390/biom16050681

**Published:** 2026-05-05

**Authors:** Dalila Khemili, Laura Carrillo-Serradell, Violeta Planells-Romeo, Lucía Aragón-Serrano, Selma Djilani, Djelila Hammoudi-Triki, Khedidja Zerouti, Abdenacer Mouffok, Francisco Lozano, María Velasco-de-Andrés

**Affiliations:** 1 Laboratory of Cellular and Molecular Biology-Tamayouz, Faculty of Biological Sciences, University of Science and Technology Houari Boumediene (USTHB), BP 32, El Alia, Bab Ezzouar, Algiers 16111, Algeria; 2 Group of Immunoreceptors of the Innate and Adaptive System, Institut d’Investigacions Biomèdiques August Pi i Sunyer (IDIBAPS), 08036 Barcelona, Spain; 3 Research and Development Laboratory, Institut Pasteur Algérie, University of Health Sciences, Algiers 16000, Algeria; 4 Faculty of Natural Sciences and Life, Department of Biology, Saad Dahlab-Blida 1 University, P.O. Box 270 Soumaa Road, Blida 09000, Algeria; 5 Laboratory of Applied Microbiology, Department of Microbiology, Faculty of Nature and Life Sciences, Setif 1 University—Ferhat ABBAS, Setif 19000, Algeria; 6 Servei d’Immunologia, Hospital Clínic de Barcelona, 08036 Barcelona, Spain; 7 Departament de Biomedicina, Facultat de Medicina, Universitat de Barcelona, 08036 Barcelona, Spain

**Keywords:** scavenger receptors, CD5, CD6, venoms, cobra, scorpion, honeybee, PLA2

## Abstract

Animal venoms induce systemic inflammatory response syndrome through their interaction, inter alia, with pattern recognition receptors (PRRs) of the innate immune system. CD5 and CD6 are lymphoid members of the scavenger receptor cysteine-rich superfamily, endowed with PRR activity against microbial-associated molecular patterns (MAMPs) derived from bacteria, fungi, viruses and/or parasites. In this study, we aimed to investigate CD5 and CD6 interaction with cobra (*Naja haje*), scorpion (*Androctonus australis*) and honeybee (*Apis mellifera*) venoms. Binding assays revealed direct, dose-dependent and specific interaction of soluble human CD5 and CD6 receptors with protein nature components from the three venoms. Proteomic analysis identified venom nerve growth factor, basic phospholipase A2 (PLA2) and cobra venom factor, in cobra venom, and scorpion venom toxins targeting potassium (α-KTx 8.1) and sodium channels (Neurotoxin-1″ and G-TI) as potentially interacting components with CD5 and CD6. Further studies confirmed direct binding of bee venom main components, phospholipase A2 and melittin, to both soluble CD5 and CD6 receptors. Interestingly, in vitro PLA2 activity from cobra and bee venom was significantly reduced by both soluble CD5 and CD6 receptors. These findings broaden the PRR properties of CD5 and CD6 and support their potential involvement in envenomation pathophysiology.

## 1. Introduction

Animal venoms are complex molecular mixtures that include polypeptide neurotoxins, cytolytic peptides, and enzymes, among others. Their toxic effects result from the synergistic action of different components on a wide array of molecular targets [1,2]. The nervous system is the principal target for animal venoms, where neurotoxins interact with ion channels, receptors, and enzymes to disrupt different stages of nerve impulse transmission [3,4].

Many envenomations are associated with severe tissue damage, owing to toxins and extracellular-matrix-degrading enzymes, mainly phospholipase A2 (PLA2) and snake venom metalloproteinases (SVMPs), which cause local and systemic tissue-damaging effects [5,6,7,8,9,10,11]. In addition to these direct toxic effects in different tissues, venoms and their toxins also impact the immune system by triggering a life-threatening systemic inflammatory response, critical in venom-induced tissue damage pathogenesis [12]. Venoms contribute to the local and systemic inflammatory events by provoking damage-associated molecular patterns (DAMPs) release from injured tissues. The recognition of venom-induced DAMPs by various TLRs such as TLR2, TLR4 and TLR9 leads to the initiation of an intense inflammatory response [13,14,15,16,17] followed by increased oxidative stress, an important aspect of venom pathogenesis, that triggers tissue damage and potentiates inflammation [18].

In most envenomations, antivenoms fail to neutralize venom-induced inflammation. Thus, understanding a venom’s inflammatory pathogenesis becomes crucial for developing inflammatory control approaches during envenomation [19]. Venoms have been described as inducers of sterile inflammation through pattern recognition receptors of the innate immune system (PRRs) involving both venom-induced DAMPs and direct recognition of venom-associated molecular patterns (VAMPs) [16,20,21]. Interestingly, the innate immune receptors TLR2, TLR4, CD14 and CD36 recognize ion channel-modulating toxins from the Brazilian scorpion *Tityus serrulatus*, resulting in proinflammatory cytokine and eicosanoid production by macrophages [22,23]. Similarly, cytokine production by *Androctonus australis* venom involves downstream signaling events of TLR engagement [24]. In response to snake envenomation, cystein-rich secretory proteins (CRISPs), known to target ion channels, have been shown to mediate inflammatory responses [25]. For instance, CRISP isolated from *Naja kaouthia* cobra venom (Nk-CRISP) engages its cysteine-rich domain (CRD) to interact with the TLR4-MD2 receptor complex, initiating proinflammatory signaling in macrophages and upregulation of several inflammatory marker genes [19]. Enzymatic toxins also appear to be recognized by innate immune receptors: L-amino acid oxidase from *Calloselasma rhodostoma* snake venom (CR-LAAO) acts by activating TLR2 and TLR4 and stimulates peritoneal macrophages to produce IL-6 and IL-1β [26]. In contrast to these activating effects, NA39, a peptide screened from the cobra *Naja atra* venom gland cDNA library, binds robustly to TLR4, preventing it from binding to MD2 and thereby modulating the activation of the TLR4 signaling pathway [27].

Similar to other PRRs, scavenger receptors (SRs) play a central role in innate immunity via a wide range of ligands, and contribute to the clearance of altered-self or non-self targets [28,29,30]. These receptors constitute a structurally heterogeneous group of transmembrane and soluble proteins. SRs are expressed on various innate immune cell types present at the portals of pathogen entry, such as macrophages, dendritic cells, neutrophils, and endothelial and epithelial cells [31,32]. However, certain SRs are exceptionally present on adaptive immune cells. This is the case of CD5 and CD6 which are expressed by all T cell types and a minor subset of B cells (B1a cells). These lymphocytic scavenger receptors are transmembrane glycoproteins organized in three tandem scavenger receptor cysteine-rich (SRCR) extracellular domains and a cytoplasmic tail that modulates activation signals delivered by the antigen-specific receptors of T and B cells (TCRs and BCRs, respectively) with which they are physically associated [30,33]. On this basis, CD5 and CD6 may exert dual immunomodulatory effects to promote immune activation while simultaneously dampening excessive inflammatory responses in different immune response contexts [34,35].

In addition to their immunomodulatory properties, CD5 and CD6 recognize a broad range of microbial-associated molecular patterns (MAMPs) from different origins. These encompass CD5 interactions with fungal β-glucans [36] and hepatitis C virus [37], while CD6 interacts with lipopolysaccharide, lipoteichoic acid and peptidoglycan of Gram-positive and Gram-negative bacteria [38] and gp120 from human immunodeficiency virus 1 [39]. Both receptors also share the ability to interact with tegumental components of the *Echinococcus granulosus* parasite [40,41]. Thus, recombinant CD5 and CD6 ectodomains have been shown to exhibit prophylactic and therapeutic potentials in fungal, bacterial and parasitic infections [30,41,42,43].

Considering the ability of certain PRRs to function as innate immune sensors for animal venoms and the diverse range of CD5 and CD6 ligands, the present study explores the PRR activity of these two lymphocytic scavenger receptors towards cobra, scorpion and honeybee venoms. The study reveals binding properties of both receptors to animal venom components. Thus, the broad-spectrum PRR activity of CD5 and CD6 is extended, beyond bacteria, fungi, viruses and parasites, to include VAMPs.

## 2. Materials and Methods

### 2.1. Venoms Collection and Toxins

Cobra venom was collected by manual milking of *Naja haje* cobra; specimens were captured from the North Center of the Sahara (Ghardaia, Algeria). Scorpion venom was obtained by electrical stimulation of *Androctonus australis* scorpions, collected in the western region of the High Plains (Ksar Chellala, Algeria). Bee venom collection was carried out using the standard electroshock method from healthy, clean colonies of local strains of *Apis mellifera*; the apiary was located in the eastern part of the High Plains (Setif, Algeria). Lyophilized *Naja haje*, *Androctonus australis* and *Apis mellifera* venoms (*Nh*V, *Aa*V and *Am*V respectively) were solubilized in sterile phosphate-buffered saline (PBS). After centrifugation at 10,000 g for 10 min at 4 °C, supernatant protein content was assessed using BCA Protein Assay Reagent (Pierce, Thermo Fisher Scientific, Rockford, IL, USA). 

Purified melittin and PLA2 from honeybee venom (*Am*V-PLA2) were purchased from Sigma-Aldrich (St. Louis, MO, USA). Crude venom and toxin (melittin and *Am*V-PLA2) aliquots were stored at −80 °C until used.

### 2.2. Expression, Purification and Biotinylation of Recombinant Proteins

Production of purified recombinant soluble proteins encompassing the whole ectodomains of human CD5 (rshCD5; from R25 to D345) and CD6 (rshCD6; from D25 to R397) receptors (in PBS with 10% glycerol, pH 7.4) was performed based on previously reported methods [44] using SURE CHO-M Cell line^TM^ clones (Selexis SUREtechnology Platform^TM^, Geneva, Switzerland) and size-exclusion chromatography protocols developed at PX’Therapeutics (Grenoble, France). Human (HSA) and bovine (BSA) serum Albumin were from Sigma-Aldrich. Proteins were biotin-labeled with EZ-Link PEO-maleimide-activated biotin (Pierce) following the manufacturer’s instructions.

### 2.3. ELISA Binding Assays

The binding ability of rshCD5 and rshCD6 proteins to cobra, scorpion and honeybee venom components was studied by ELISA, following a previously published protocol [36]. Briefly, 96-well microtiter plates (Nunc, Roskilde, Denmark) were coated overnight at 4 °C with 100 μL/well of cobra, scorpion or honeybee venom at 5 μg/mL in PBS. The plates were then blocked for 1 h at room temperature (RT) with 200 μL/well of PBS containing 1% (*w*/*v*) BSA. Increasing concentrations (0–10 μg/mL) of biotin-labeled rshCD5, rshCD6 or HSA were added to the wells (100 μL/well, triplicates) and incubated for 2 h at RT. Bound protein was detected by the addition of HRP-conjugated streptavidin (100 μL/well; 1:5000; Sigma) for 1 h at RT. Following each incubation step, unbound proteins were washed out thrice with PBS containing 0.05% (*v*/*v*) Tween-20. The ELISA was developed by adding 3,3′,5,5′-tetramethylbenzidine liquid substrate (TMB; BD OptEIA^TM^) for 30 min at RT prior to stopping the reaction with H_2_SO_4_ (0.5 M; 50 μL/well). Absorbance was read at 450–570 nm.

A urea dissociation test was performed to break the low-affinity interactions between venom components and biotin-labeled proteins, thereby preventing the occurrence of unspecific cross-reactivity [45,46]. The assay consists of dissociating agent addition (6 M urea in PBS containing 0.05% (*v*/*v*) Tween-20; 200 μL/well) for 20 min at RT after the incubation period with biotin-labeled proteins and prior to HRP-conjugated streptavidin addition. The remaining ELISA protocol was performed as described above.

Competitive ELISA assays were performed as detailed above with a preincubation step of biotin-labeled proteins (0.625 µg/mL), for 1 h at RT, with increasing concentrations of zymosan for rshCD5, and lipopolysaccharide (LPS, purified from *E. coli* O55:B5, Sigma), lipoteichoic acid (LTA; Sigma) or peptidoglycan (PGN; Sigma) for rshCD6, before their addition to venom-coated plates.

To verify the protein nature of rshCD5 and rshCD6 interactors present in venoms, a GuHCl-modified ELISA assay was performed including a protein denaturation step with guanidium chloride [47]. Venom-coated and BSA-blocked plates were treated with guanidine hydrochloride (GuHCl) (8 M in PBS containing 0.05% (*v*/*v*) Tween-20; 200 μL/well) for 20 min at RT. PBS-Tween-20 without GuHCl was added to control wells. The plates were washed thrice and the remainder of the assay is the same as the above-detailed ELISA assay. rshCD5 and rshCD6 binding to GuHCl-treated venom components was expressed as the percentage of absorbance values in GuHCl-treated wells related to the absorbance of untreated wells.

To explore potential overlapping between rshCD5 and rshCD6 for their interaction with venoms, competitive ELISA was carried out using unlabeled and biotin-labeled proteins. Venom-coated and BSA-blocked plates were incubated with biotin-labeled rshCD5 or rshCD6 (0.625 µg/mL) and increasing concentrations of unlabeled rshCD6 or rshCD5 (0–10 μg/mL) respectively. Results are expressed as percentage of absorbance values in competed wells related to non-competed wells.

### 2.4. Sodium Dodecyl Sulfate Polyacrylamide Gel Electrophoresis (SDS-PAGE) Analysis

Venoms were analyzed by 15% SDS-PAGE under reducing conditions, according to the Laemmli method [48]. Venoms were mixed with sample buffer (10% glycerol, 50 mM Tris-HCl, pH 6.8, 2% (*w*/*v*) SDS and 0.1% bromophenol blue) containing 6% 2-mercaptoethanol (2-ME). The mixture was denatured for 5 min in 100 °C and then subjected to electrophoresis at 190 V for 1 h in a Bio-Rad system in addition to molecular weight markers (10–180 kDa). After sample resolution, migrated venom proteins were analyzed by Western blot to assess their binding to rshCD5 and rshCD6. In parallel, gels were stained using Coomassie Brilliant Blue R250 (SERVA Electrophoresis GmbH, Heidelberg, Germany)

### 2.5. Native Polyacrylamide Gel Electrophoresis (Native-PAGE) Analysis

The binding ability of rshCD5 and rshCD6 proteins to *Naja haje* cobra venom (*Nh*V) was assessed after electrophoretic separation of venom components in native conditions. In brief, venom was mixed with native-PAGE sample buffer (10% glycerol, 50 mM Tris-HCl, pH 6.8, and 0.1% bromophenol blue). Venom samples (90, 150, 180 and 200 µg) were loaded in a continuous 10% native polyacrylamide gel [49], and then subjected to electrophoresis at 150 V for 1 h in a Bio-Rad system. Following samples resolution, migrated venom proteins were analyzed by Western blot to assess their binding to rshCD5 and rshCD6. In parallel, gels were stained using Coomassie Brilliant Blue R250.

### 2.6. Western Blotting

Native-PAGE and SDS-PAGE gels containing the migrated venom proteins were electro-transferred to nitrocellulose membranes (GE Healthcare life Sciences, Chicago, IL, USA), and blocked with 5% skim milk (*w*/*v*, in TBS-Tween 20) for 2 h at 4 °C. Membranes were incubated for 2 h at 4 °C with biotin-labeled rshCD5, rshCD6 or HSA (30 µg/mL), followed by incubation with HRP-conjugated streptavidin (1:2000; Sigma) for 1 h at RT. After membrane washing with TBS-Tween20, blots were developed using Amersham^TM^ ECL Western Blotting Detection Reagent (Cytiva, Amersham, UK) in a G-Box (Syngene, Cambridge, UK).

### 2.7. Proteomic Analysis

For in-gel digestion, bands of interest were excised and washed with ammonium bicarbonate (50 mM) and acetonitrile (ACN). Proteins were reduced (dithiothreitol 20 mM; 60 min, 56 °C) and alkylated (iodoacetamide 55 mM; 25 °C, 30 min, in the dark). Afterwards, proteins were digested for 2 h with trypsin (sequencing grade modified Trypsin, Promega, Madison, WI, USA, pH 8, 37 °C) and were digested overnight with the same enzyme. The resulting peptide mixtures were extracted from the gel matrix with 5% formic acid solution (5% FA/H_2_O) and 100% ACN, dried down in a SpeedVac vacuum system and stored at −20 °C until liquid chromatography–mass spectrometry (LC-MS) analysis.

The dried-down peptide mixtures were analyzed in a nanoAcquity liquid chromatographer (Waters) coupled to an LTQ-Orbitrap Velos (Thermo Scientific) mass spectrometer. The tryptic digests were resuspended in 1% FA solution and an aliquot per sample was injected for chromatographic separation. Peptides were trapped on a Symmetry C18TM trap column (5 μm, 180 μm × 20 mm; Waters, MA, USA) and were separated using a C18 reverse phase capillary column (biozenTM peptide XB-C18 column; 2.6 μm, 75 μm × 250 mm; Phenomenex, Torrance, CA, USA). The gradient used for the elution of the peptides was 1 to 40% B in 30 min, followed by a gradient from 40 to 60% in 5 min. (A: 0.1% FA; B: 100% ACN, 0.1% FA; flow rate: 300 nL/min).

Eluted peptides were subjected to electrospray ionization in an emitter needle (Thermo) with an applied voltage of 2100 V. Peptide masses (*m*/*z* 300–1600) were analyzed in data-dependent mode where a full Scan MS was acquired in the Orbitrap with a resolution of 60,000 FWHM at 400 *m*/*z*. Up to the 15th most abundant peptides (minimum intensity of 500 counts) were selected from each MS scan and then fragmented in the linear ion trap using CID (38% normalized collision energy) with helium as the collision gas. The scan time settings were: full MS: 250 ms (1 microscan) and MSn: 120 ms. Generated raw data files were collected with ThermoXcalibur (v.2.2).

The raw data files obtained in the mass spectrometry analyses were used to search against modified versions of the public database SwissProt which were merged with the TrEMBL database for scorpions and serpents. In addition, data searches were also performed against modified versions of the Uniprot databases for scorpions and serpents, which were merged with a small database containing laboratory contaminants. Database searches were performed with the Sequest HT search engine using Thermo Proteome Discover (v.1.4.1.14).

### 2.8. Phospholipase A2 Activity Assay

PLA2 activity was assayed by the turbidimetric method following slightly modified protocols [50,51]. Before PLA2 assay, cobra venom (*Nh*V at 50 µg/mL) or phospholipase A2 from honeybee venom (*Am*V-PLA2 at 5 µg/mL) were preincubated, for 1 h at RT, with rshCD5 or rshCD6 at different concentrations to obtain different ratios of *Nh*V or *Am*V-PLA2 to soluble receptors. For assaying PLA2 activity, 50 µL of different ratios were mixed with 150 µL of reaction mixture (egg yolk suspended in 0.9% (*w*/*v*) NaCl containing 0.02% (*w*/*v*) sodium azide and diluted in 0.1 M Tris-HCl buffer, pH 8.0). The decrease in turbidity after 10 min was monitored at 740 nm against a reagent blank. One unit of PLA2 activity has been arbitrarily defined as a decrease in 0.01 absorbance at 740 nm after 10 min of incubation. The results are indicated as residual activity percentage, where 100% corresponds to the activity induced by *Nh*V or *Am*V-PLA2 alone.

### 2.9. Statistical Analysis

GraphPad Prism v.5 software was used for statistical analysis. Data are presented as mean ± standard error of the mean (SEM) and statistical significance between data groups was evaluated using one-way ANOVA and Tukey’s multiple comparison test. Differences were considered statistically significant when *p* < 0.05.

## 3. Results

### 3.1. Soluble CD5 and CD6 Ectodomains Bind to Venoms Components

**Figure 1:**
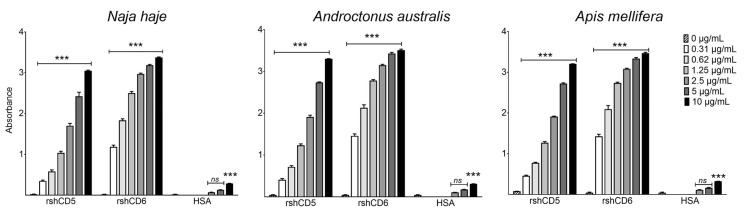
Binding of CD5 and CD6 ectodomains to cobra, scorpion and honeybee venom components. ELISA assays showing the binding of increasing concentrations of biotin-labeled rshCD5, rshCD6 and HSA to *Nh*, *Aa* and *Am* venom-coated plates (5 µg/mL). Data are expressed as mean ± SEM (*n* = 3) and statistical significances over control were assessed by one-way ANOVA and Tukey’s multiple comparison test. ns, *p* > 0.05; ***, *p* < 0.001.

The possibility of rshCD5 and rshCD6 interacting with components of *Naja haje* cobra, *Androctonus australis* scorpion and *Apis mellifera* honeybee venoms (*Nh*V, *Aa*V and *Am*V respectively) was first explored by ELISA-based binding assays. Increasing concentrations of biotin-labeled rshCD5, rshCD6 and HSA proteins were tested on venom-coated ELISA plates. The results in Figure 1 disclose dose-dependent binding ability of both rshCD5 and rshCD6 with molecules present in the three tested venoms.

**Figure 2:**
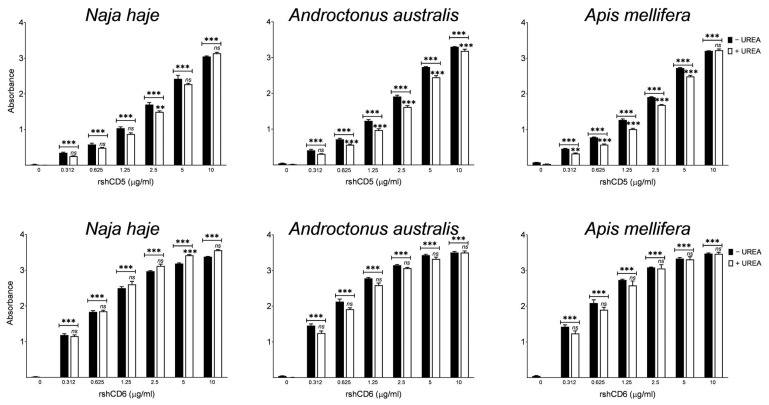
Binding of CD5 and CD6 ectodomains to cobra, scorpion and honeybee venom components in the presence of urea. Binding of increasing concentrations of biotin-labeled rshCD5 and rshCD6 to *Nh*, *Aa* and *Am* venom-coated plates (5 µg/mL) was performed in the presence or absence of urea (6 M). Data are expressed as mean ± SEM (*n* = 3) and statistical significances were assessed by one-way ANOVA and Tukey’s multiple comparison test. ns, *p* > 0.05; **, *p* < 0.01; ***, *p* < 0.001 compared to control and urea-untreated wells.

The same binding assays were performed in the presence of urea used as dissociating and denaturing agent to alleviate low-affinity or false-positive interactions [52]. As shown in Figure 2, the absorbance values obtained in the presence of urea remained significantly higher compared to the control despite an overall decrease. Thus, CD5 and CD6 ectodomains seem to bind to venoms with relative avidity.

**Figure 3:**
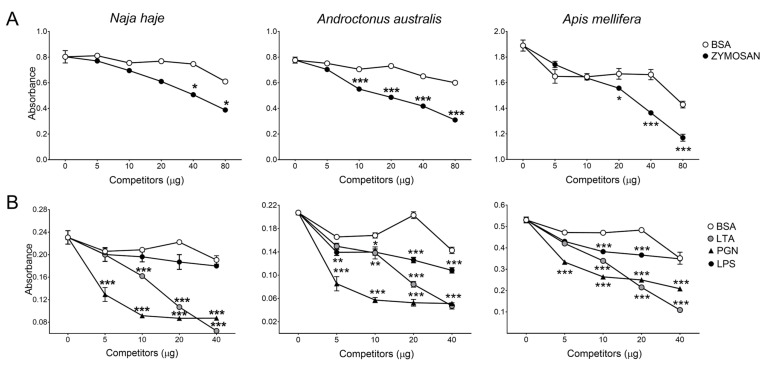
Competition of CD5 and CD6 ectodomain binding to venom components by MAMPs. A fixed concentration of biotin-labeled proteins (0.625 µg/mL) was incubated with venom-coated plates (5 µg/mL) in the absence or the presence of increasing amounts of unlabeled competitors: zymosan or BSA for rshCD5 (**A**) and LPS, LTA, PGN or BSA for rshCD6 (**B**). Data are expressed as mean ± SEM (*n* = 3) and statistical significances were assessed by one-way ANOVA and Tukey’s multiple comparison test. *, *p* < 0.05; **, *p* < 0.01; ***, *p* < 0.001 compared to BSA-competed wells.

Competition ELISA assays were next performed in which the binding of CD5 and CD6 ectodomains to venoms was competed in the presence of MAMPs previously reported to interact with both receptors. The results depicted in Figure 3 show that zymosan competed the binding of rshCD5 to *Nh*V, *Aa*V and *Am*V components. Likewise, LPS, LTA and PGN showed dose-dependent competition with the three venom components to bind with rshCD6.

**Figure 4:**
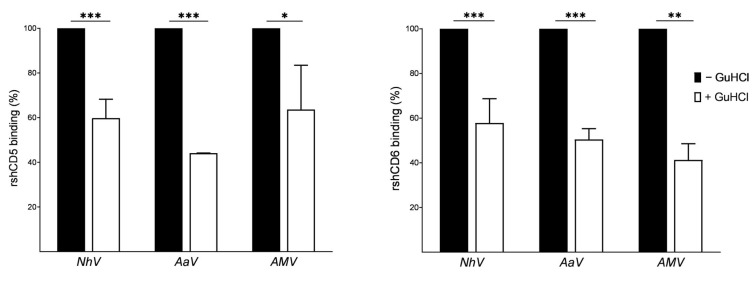
Binding of CD5 and CD6 ectodomains to venom components in the presence of guanidine hydrochloride. GuHCl-modified ELISA assays showing the binding of biotin-labeled rshCD5 or rshCD6 (0.625 µg/mL) to *Nh*, *Aah* and *Am* venom-coated plates after protein denaturation with guanidine hydrochloride (GuHCl). Data are expressed as mean ± SEM (*n* = 3) and statistical significances were assessed by one-way ANOVA and Tukey’s multiple comparison test. *, *p* < 0.05; **, *p* < 0.01; ***, *p* < 0.001 compared to GuHCl untreated wells.

Since venoms are complex mixtures of protein and non-protein components, ELISA assays were carried out in the presence of guanidine hydrochloride (a chaotropic, protein-denaturing agent) to ascertain the protein nature of the venom components bound to CD5 and CD6. From the results of the GuHCl-modified ELISA assays in Figure 4, the binding of biotin-labeled rshCD5 and rshCD6 is significantly reduced after protein denaturation, suggesting that both receptors interact with protein components of *Nh*V, *Aa*V and *Am*V.

**Figure 5:**
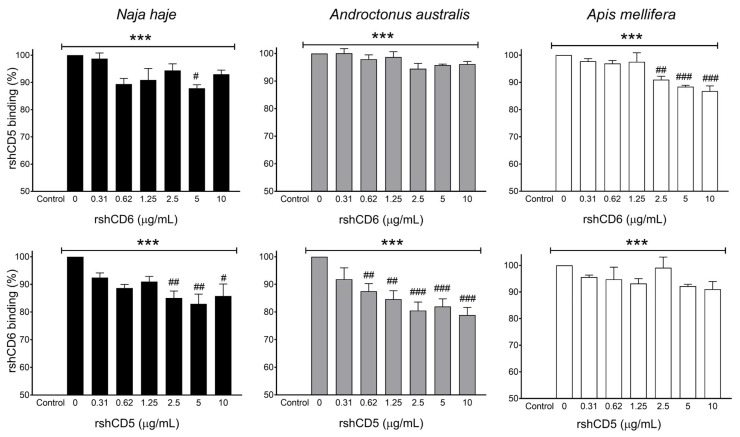
Competitive binding between CD5 and CD6 to venom components. Competitive ELISA assays showing the binding of a fixed concentration of biotin-labeled rshCD5 or rshCD6 to venom-coated plates (5 µg/mL) in competition with increasing concentrations of unlabeled rshCD6 or rshCD5, respectively. Data are expressed as mean ± SEM (*n* = 3) and statistical significances were assessed by one-way ANOVA and Tukey’s multiple comparison test. ***, *p* < 0.001 compared to control. #, *p* < 0.05; ##, *p* < 0.01; ###, *p* < 0.001 compared to non-completed wells.

The potential overlapping between CD5 and CD6 in their interaction with venom components was explored by competitive ELISA, using a fixed concentration of biotin-labeled rshCD5 or rshCD6 and increasing concentrations of unlabeled rshCD6 or rshCD5 respectively. Results in Figure 5 illustrate rshCD5 and rshCD6 overlapping binding abilities to *Nh*V, *Aa*V and *Am*V, suggesting similarity of ligand patterns between CD5 and CD6 ectodomains.

### 3.2. CD5 and CD6 Ectodomains Bind to the Two Main Components of Bee Venom

**Figure 6:**
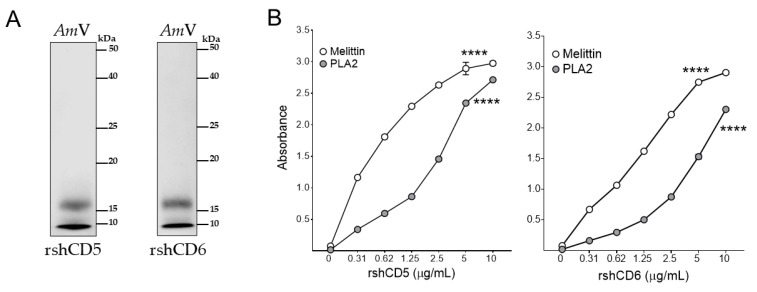
Interaction of melittin and phospholipase A2 from *Apis mellifera* venom (*Am*V-PLA2) with CD5 and CD6 ectodomains. (**A**) Western blotting of *Apis mellifera* venom proteins (10 µg) by SDS-PAGE under reducing conditions (+βME). Immunoblots were developed with biotin-labeled rshCD5 or rshCD6 (30 µg/mL). (**B**) ELISA assays showing direct binding of increasing concentrations of biotin-labeled rshCD5 and rshCD6 to melittin and *Am*V-PLA2 coated plates (5 µg/mL). Data are expressed as mean ± SEM (*n* = 3) and statistical significances were assessed by one-way ANOVA and Tukey’s multiple comparison test. ****, *p* < 0.0001 compared to control. Original western blots can be found at Supplementary Materials.

In an attempt to assess which venom components interact with CD5 and CD6, Western blot analysis of *Am*V proteins separated by reducing SDS-PAGE were performed. As shown in Figure 6A, biotin-labeled rshCD5 and rshCD6 revealed two *Am*V protein bands of ≈15 kDa and <10 kDa, in agreement with bee venom high- and low-molecular-weight protein components (1–88 kDa), such as enzymes, peptides, and vasoactive amines [53,54].

Direct binding of CD5 and CD6 ectodomains to purified honeybee venom components was further assessed by ELISA. Melittin and PLA2 (*Am*V-PLA2), the most prevalent protein components in bee venom, constitute 40–60% and 12–15% of the venom’s dry weight, respectively [55]. Figure 6B shows dose-dependent rshCD5 and rshCD6 binding to melittin and *Am*V-PLA2. This result identifies melittin and *Am*V-PLA2 as ligands of CD5 and CD6. Whether CD5 and CD6 may interact with other minor components of bee venom such as apamin, mast cell degranulating (MCD) peptide, adolapine, secapin and procamine still awaits confirmation.

### 3.3. CD5 and CD6 Ectodomains Bind to Toxins in Cobra and Scorpion Venoms

Bioactive proteins and polypeptides in snake venoms are known to form covalent and non-covalent complexes. These higher-order structures exhibit more potent pharmacological activities compared to individual components and play an important role in the pathophysiology of envenomation [56,57,58,59,60,61].

**Figure 7:**
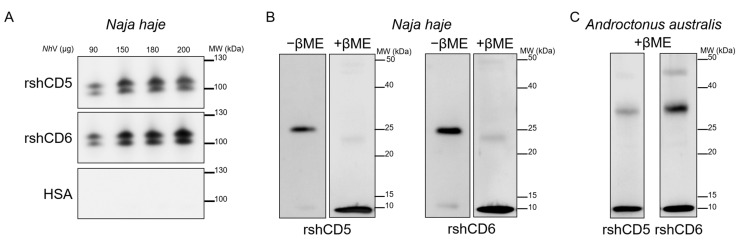
Interaction of CD5 and CD6 ectodomains with low-molecular-weight cobra and scorpion venom components. (**A**) Immunoblots showing the binding of increasing amounts of *Naja haje* venom (90, 150, 180 and 200 µg) subjected to native-PAGE, and developed with biotin-labeled rshCD5, rshCD6 or HSA (30 µg/mL). (**B**) Western blotting *Naja haje* venom proteins (10 µg) subjected to SDS-PAGE under reducing (+βME) and non-reducing (−βME) conditions. (**C**) Western blotting *Androctonus australis* venom proteins (10 µg) subjected to SDS-PAGE under reducing conditions (+βME) and further developed with biotin-labeled rshCD5 or rshCD6 (30 µg/mL). Original western blots can be found at Supplementary Materials.

Consequently, the binding of CD5 and CD6 ectodomains to cobra (*Naja haje*) venom components was further analyzed by Western blotting under native, reducing and non-reducing conditions. According to Figure 7A, native-PAGE and further Western blotting of increasing amounts of crude *Nh*V (90, 150, 180 and 200 µg) against biotin-labeled rshCD5 or rshCD6 revealed dose-dependent binding of CD5 and CD6 to two *Nh*V protein bands of ≥100 kDa. Western blot analysis of *Nh*V proteins separated by SDS-PAGE under non-reducing conditions revealed binding of CD5 and CD6 to only one *Nh*V protein band of ≈25 kDa. Under reducing conditions CD5 and CD6 interact with one *Nh*V protein band of ≈10 kDa (Figure 7B). Molecular weight differences of the *Nh*V interacting protein bands between native, reduced and non-reduced gels respond to protein complex dissociation and reveal non-covalent and covalent protein complexes in *Naja haje* cobra venom. Furthermore, these data provide evidence of CD5 and CD6 binding to *Nh*V native components within non-covalent and/or covalent protein complexes, with sequence and conformational motifs contributing to these interactions.

Interestingly, Western blot analysis of scorpion (*Androctonus australis*) venom proteins (*Aa*V) separated by SDS-PAGE under reducing conditions showed that CD5 and CD6 also bind to two *Aa*V protein bands of ≈30 kDa and ≈10 kDa (Figure 7C).

Bands of interest from Coomassie Blue-stained PAGE from cobra and scorpion venoms were further subjected to LC-MS/MS spectrometry to identify proteins that may potentially interact with CD5 and CD6. Three bands of interest from cobra venom, two that interacted with CD5 and CD6 in native-PAGE and one in reducing SDS-PAGE, identified three proteins: venom nerve growth factor, basic phospholipase A2 and cobra venom factor (Table 1).

Analysis of the two interactive bands from scorpion venom SDS-PAGE simultaneously revealed three toxins as potential ligands for CD5 and CD6: the potassium channel toxin α-KTx 8.1, and the sodium channel toxins Neurotoxin-1″ and G-TI (Table 1).

### 3.4. CD5 and CD6 Ectodomains Interfere with In Vitro Venom Phospholipolytic Activity

**Figure 8:**
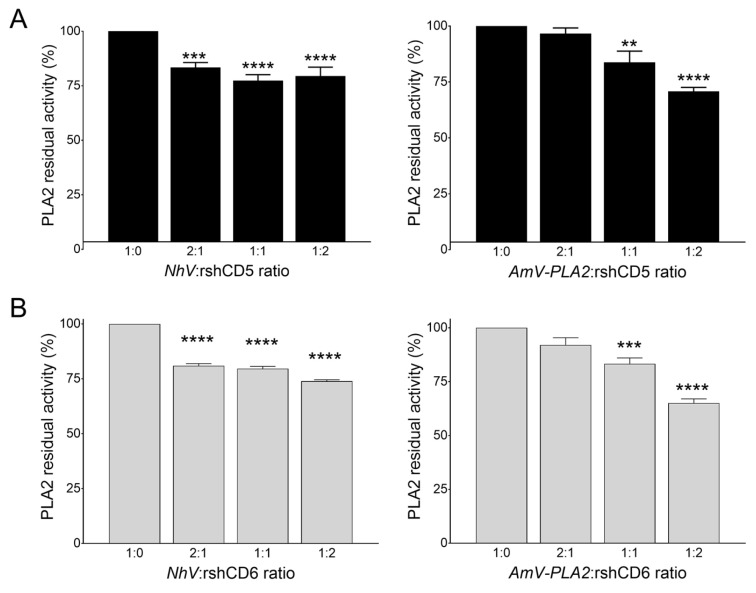
CD5 and CD6 ectodomains interfere with the PLA2 activity of *Naja haje* and *Apis mellifera* venoms. Residual PLA2 activity of *Naja haje* and *Apis mellifera* venom in the presence or absence of rshCD5 (**A**) or rshCD6 (**B**) at the indicated ratios. Data are expressed as mean ± SEM (*n* = 4) and statistical significances were assessed by one-way ANOVA and Tukey’s multiple comparison test. **, *p* < 0.01; ***, *p* < 0.001; ****, *p* < 0.0001 compared to control.

In order to check whether the interaction of CD5 and CD6 with PLA2 interferes with its catalytic activity, PLA2 activity assays were performed. To that end, the phospholipolytic activity of cobra (*Nh*V) and honeybee (*Am*V) venom PLA2 was assessed in the presence of rshCD5 or rshCD6 at *Nh*V- or *Am*V-PLA2:soluble receptor ratios of 1:0, 2:1, 1:1 and 1:2. As shown in Figure 8, *Nh*V (50 µg/mL) exhibited reduced PLA2 enzymatic activity for all tested concentrations. In addition, 76 ± 4% and 73.9 ± 0.6% of PLA2 residual activity were obtained when *Nh*V was preincubated with rshCD5 or rshCD6 (ratio 1:2) respectively (*n* = 4, *p* < 0.0001). Similarly, enzymatic activity of *Am*V-PLA2 (5 µg/mL) was found to decrease in the presence of increasing concentrations of both proteins as can be seen in Figure 8. rshCD5 and rshCD6 with *Am*V-PLA2:soluble receptor ratio of 1:2 resulted respectively in 67.4 ± 1.7% and 65.1 ± 1.9% of residual activity (*n* = 4, *p* < 0.0001). These findings confirm that PLA2 in cobra venom is a ligand for CD5 and CD6. The partial inhibition of PLA2 activity could be explained by steric hindrance following CD5 and CD6 binding, probably hampering substrate access to the active site of PLA2 [68].

## 4. Discussion

Animal venoms contain potent inducers of tissue damage and inflammatory response. The innate immune system detects venoms through their recognition by a variety of PRRs, including TLRs (TLR2 and TLR4) [19,22,24,26], the mannose receptor (CD206) [69,70] and the B2 scavenger receptor (CD36) [23]. The present findings provide the first evidence of the lymphocytic scavenger receptors CD5 and CD6 as newcomers to the list of PRRs that sense animal venom components, as shown for three different animal species: the cobra *Naja haje*, the scorpion *Androctonus australis* and the honeybee *Apis mellifera*.

Animal toxins are characterized by exquisite pharmacological diversity, specificity and selectivity that affect different physiological processes. Scorpion envenomation, mediated by ion channel-modulating toxins, induces a “neurotransmitter storm” and triggers a wide spectrum of symptoms, ranging from severe local skin reaction to multiple organ failure, accompanied by systemic inflammatory response syndrome (SIRS) in severe cases [12]. Three different toxins from *Androctonus australis* scorpion venom are identified as potential ligands for CD5 and CD6 ectodomains: α-KTx 8.1 acting on voltage-gated potassium channels Kv1.3 and Kv1.1, and Neurotoxin-1″ and G-TI, which are selective and high-affinity ligands for the voltage-gated sodium channels (Na_V_). These toxins are mono-concatenated peptides, adopting a tight tridimensional-shaped backbone, a conserved cysteine-stabilized α-helix/β-sheet (CSαβ) motif containing three antiparallel β-strands and an α-helix cross-linked by 3–4 disulfide bridges [71]. Sensing of scorpion neurotoxins by PRRs has been reported for the sodium channel β-neurotoxin Ts1, the major toxin component of *Tityus serrulatus* venom [22]. Scorpion neurotoxins are also potent immune system modulators, involving indirect stimulation through the neuroendocrine–immune axis, direct interaction with ion channels and transporters expressed in immune cells and direct activation of innate immune receptors [20,21,72,73,74]. The toxicity of *Androctonus* venom is largely ascribed to the long-chain toxins that act as gating modifiers of Na_V_ channels, with α-toxins being the main peptides responsible for its lethal effect [75]. Notably, the identification of Neurotoxin-1″ as a ligand of CD5 and CD6 is particularly striking, as this toxin is one of the three α-toxins responsible for the majority of *Androctonus australis* venom toxicity in mammals [76].

In cobra venom, the three different proteins identified as potential ligands of CD5 and CD6 include: venom nerve growth factor (vNGF), basic phospholipase A2 (bPLA2) and cobra venom factor (CVF), the complement-activating protein [77,78]. Among these, bPLA2 (by similarity to svPLA2s) plays the most relevant role in snakebite envenomation. In fact, svPLA2s are key mediators of venom toxicity, contributing to diverse pharmacological mechanisms that include neurotoxicity, myotoxicity, hemorrhage, edema, cardiotoxicity, and tissue damage [79]. In *Elapidae* venoms, svPLA2s are the second major constituent, representing an average of 27% of the whole venom proteome [80]. Of particular concern are several species of cobra native to Africa, especially the cobra *Naja haje*, widespread from southern Egypt to northern South Africa. *Naja haje* venom is highly potent and characterized by rapidly diffusing three-finger toxins (3FTxs) that induce fatal respiratory paralysis. This effect is mainly attributed to 3FTxs binding to postsynaptic nicotinic acetylcholine receptors (nAChRs), disrupting the nerve signal transmission and subsequently leading to skeletal muscle paralysis [81,82]. This paralysis can extend to the diaphragm, leading to respiratory failure [83]. However, much evidence points to the contribution of svPLA2s in this life-threatening neuromuscular paralysis. These β-neurotoxins mediate a multi-site synaptic transmission failure, via depletion of presynaptic neurotransmitter vesicles, inactivation of nAChRs and eventual physical degeneration of the neuromuscular endplate [84,85]. Moreover, svPLA2s act synergistically with the cytotoxic 3FTxs to produce local and systemic skeletal muscle degeneration [8]. In addition to the aforementioned effects, svPLA2s trigger venom-induced inflammation, acting directly on phospholipid membranes to release arachidonic acid, which in turn activates macrophages and other cells to produce inflammatory mediators [86,87,88,89]. Importantly, CD5 and CD6 ectodomains were able to reduce in vitro phospholipolytic activity of *Naja haje* cobra venom, suggesting a modulatory role of these receptors in venom-induced toxicity.

Interestingly, PLA2s are integral and conserved components of venoms from divergent animal species, including insects, arachnids and reptiles [90]. In bee venom, this enzyme is the most immunogenic protein and the major allergen that induces immunoglobulin E (IgE)-mediated anaphylaxis [91]. In addition to phospholipid hydrolysis, PLA2-induced degranulation of mast cells, coupled with histamine and serotonin release, induces inflammatory response and edema [92,93]. Melittin, a highly hemolytic and cytotoxic peptide, enhances PLA2 enzymatic activity leading to further phospholipid hydrolysis [53]. CD5 and CD6 were able to bind to melittin in addition to binding PLA2 from honeybee venom (*Am*V-PLA2) and to reduce its enzymatic activity in vitro. The ability of the innate immune system to detect the activity of a conserved component of venoms has previously been reported, notably bee venom PLA2, which results in induction of a primary type 2 response that confers a protective immune response against this venom toxin [94]. Moreover, it was previously demonstrated that bee venom PLA2 can be sensed by the innate immune system through its binding to CD206 mannose receptor on macrophages and dendritic cells [69,70].

## 5. Conclusions

Our findings provide novel evidence on the ability of the lymphocytic scavenger receptors CD5 and CD6 to sense venom components, attributable to several extracellular SRCR repeats, ancient and highly conserved domains of the innate immune system. Both paralog receptors exhibit previously reported binding properties to MAMPs, which can be now extended to VAMPs. They both show venom binding activity towards scorpion ion channel neurotoxins, melittin and PLA2. Moreover, binding to the conserved noxious component of cobra and honeybee venoms results in reduction in PLA2 enzymatic activity. These data provide new insight into the mechanisms by which the innate immune system contributes to sensing and scavenging animal toxins. However, further work is warranted to explore the capacity of these receptors to interfere with the venom’s pharmacological and inflammatory effects.

## Figures and Tables

**Table 1:** Potential ligands from *Naja haje* and *Androctonus australis* venoms for rshCD5 and rshCD6, identified by LC-MS/MS analysis.

Potential Ligand	Accession Number *	Species	Score	Coverage [%]	# AAs	MW [kDa]	Calc. pI	Molecular Function	Refs.
*Naja haje* venom									
Venom nerve growth factor	P61898	*Naja atra*	1874.22	97.41	116	13.1	6.51	Nerve growth factor	[62,63]
Basic phospholipase A2	P00599	*Naja melanoleuca*	171.70	41.53	118	13.5	7.42	Phosphatidylcholine 2-acid hydrolase activity	[62,63,64]
Cobra venom factor	Q91132	*Naja kaouthia*	136.31	19.12	1642	184.4	6.40	Complement-activating protein	[63]
*Androctonus australis* venom									
Potassium channel toxin alpha-KTx 8.1	P56215	*Androctonus mauritanicus mauritanicus*	43.88	100.00	29	3.2	4.72	Potassium-channel-impairing toxin	[65]
Neurotoxin-1″	P01479	*Androctonus australis*	154.53	71.08	83	9.1	8.12	Alpha-voltage-gated sodium-channel-impairing toxin	[66]
G-TI	E6ZB76	*Androctonus australis garzonii*	192.13	40.23	87	9.5	7.58	Sodium channel inhibitor activity	[67]

* Accession number of the protein in UniProt database. Score: the sum of the scores of the individual peptides. Coverage: the percentage of the protein sequence covered by identified peptides. # AAs: the sequence length of the protein. MW: the calculated molecular weight of the protein. Calc. pI: the theoretically calculated isoelectric point.

## Data Availability

The original contributions presented in the study are included in the article; further inquiries can be directed to the corresponding authors.

## Supplementary Materials

The following supporting information can be downloaded at: https://www.mdpi.com/article/10.3390/biom16050681/s1, Original western blots can be found in this section.

## References

[1] Inserra M.C., Lewis R.J. (2011). Venom peptide modulators of the immune system. Inflamm. Allergy-Drug Targets (Former. Curr. Drug Targets-Inflamm. Allergy) (Discontin.).

[2] Minutti-Zanella C., Gil-Leyva E., Vergara I. (2021). Immunomodulatory properties of molecules from animal venoms. Toxicon.

[3] Ayvazyan N.M., O’Leary V.B., Dolly J.O., Ovsepian S.V. (2019). Neurobiology and therapeutic utility of neurotoxins targeting postsynaptic mechanisms of neuromuscular transmission. Drug Discov. Today.

[4] Pei S., Wang N., Mei Z., Zhangsun D., Craik D.J., McIntosh J.M., Zhu X., Luo S. (2024). Conotoxins targeting voltage-gated sodium ion channels. Pharmacol. Rev..

[5] Ami A., Oussedik-Oumehdi H., Laraba-Djebari F. (2017). Biochemical and biological characterization of a dermonecrotic metalloproteinase isolated from Cerastes cerastes snake venom. J. Biochem. Mol. Toxicol..

[6] Dahdouh F., Belhamzaoui K., Aouadi L., Aldahmash W., Harrath A.H., Plavan G., Smaali M.E.-A., Djebar-Berrabah H. (2023). Bee venom causes oxidative stress, biochemical and histopathological changes in the kidney of mice. Physiol. Res..

[7] Gutiérrez J.M., Rucavado A. (2000). Snake venom metalloproteinases: Their role in the pathogenesis of local tissue damage. Biochimie.

[8] Gutiérrez J.M., Ownby C.L. (2003). Skeletal muscle degeneration induced by venom phospholipases A2: Insights into the mechanisms of local and systemic myotoxicity. Toxicon.

[9] Nourreddine F.Z., Oussedik-Oumehdi H., Laraba-Djebari F. (2020). Myotoxicity induced by *Cerastes cerastes* venom: Beneficial effect of heparin in skeletal muscle tissue regeneration. Acta Trop..

[10] Ownby C.L., Fletcher J.E., Colberg T.R. (1993). Cardiotoxin 1 from cobra (*Naja naja atra*) venom causes necrosis of skeletal muscle in vivo. Toxicon.

[11] Teixeira A., Fontoura B., Freire-Maia L., Machado C., Camargos E., Teixeira M. (2001). Evidence for a direct action of *Tityus serrulatus* scorpion venom on the cardiac muscle. Toxicon.

[12] Teixeira C., Moreira V., Gutiérrez J.M. (2017). Venoms. Inflamm. Mol. Cell. Mech. Clin..

[13] Chakrabartty S., Alam M.I., Bhagat S., Alam A., Dhyani N., Khan G.A., Alam M.S. (2019). Inhibition of snake venom induced sterile inflammation and PLA2 activity by Titanium dioxide Nanoparticles in experimental animals. Sci. Rep..

[14] Fontana B.C., Soares A.M., Zuliani J.P., Gonçalves G.M. (2022). Role of Toll-like receptors in local effects in a model of experimental envenoming induced by *Bothrops jararacussu* snake venom and by two phospholipases A2. Toxicon.

[15] Khemili D., Laraba-Djebari F., Hammoudi-Triki D. (2020). Involvement of toll-like receptor 4 in neutrophil-mediated inflammation, oxidative stress and tissue damage induced by scorpion venom. Inflammation.

[16] Moreira V., Teixeira C., Da Silva H.B., Lima M.R.D.I., Dos-Santos M.C. (2016). The role of TLR2 in the acute inflammatory response induced by *Bothrops atrox* snake venom. Toxicon.

[17] Zornetta I., Caccin P., Fernandez J., Lomonte B., Gutierrez J.M., Montecucco C. (2012). Envenomations by *Bothrops* and *Crotalus* snakes induce the release of mitochondrial alarmins. PLoS Neglected Trop. Dis..

[18] Resiere D., Mehdaoui H., Neviere R. (2022). Inflammation and oxidative stress in snakebite envenomation: A brief descriptive review and clinical implications. Toxins.

[19] Deka A., Sharma M., Mukhopadhyay R., Devi A., Doley R. (2020). *Naja kaouthia* venom protein, Nk-CRISP, upregulates inflammatory gene expression in human macrophages. Int. J. Biol. Macromol..

[20] Reis M.B., Zoccal K.F., Gardinassi L.G., Faccioli L.H. (2019). Scorpion envenomation and inflammation: Beyond neurotoxic effects. Toxicon.

[21] Ryan R.Y., Seymour J., Loukas A., Lopez J.A., Ikonomopoulou M.P., Miles J.J. (2021). Immunological responses to envenomation. Front. Immunol..

[22] Zoccal K.F., Bitencourt C.d.S., Paula-Silva F.W.G., Sorgi C.A., de Castro Figueiredo Bordon K., Arantes E.C., Faccioli L.H. (2014). TLR2, TLR4 and CD14 recognize venom-associated molecular patterns from *Tityus serrulatus* to induce macrophage-derived inflammatory mediators. PLoS ONE.

[23] Zoccal K.F., Gardinassi L.G., Sorgi C.A., Meirelles A.F., Bordon K.C., Glezer I., Cupo P., Matsuno A.K., Bollela V.R., Arantes E.C. (2018). CD36 shunts eicosanoid metabolism to repress CD14 licensed interleukin-1β release and inflammation. Front. Immunol..

[24] Saidi H., Bérubé J., Laraba-Djebari F., Hammoudi-Triki D. (2018). Involvement of alveolar macrophages and neutrophils in acute lung injury after scorpion envenomation: New pharmacological targets. Inflammation.

[25] Bernardes C.P., Menaldo D.L., Zoccal K.F., Boldrini-Franca J., Peigneur S., Arantes E.C., Rosa J.C., Faccioli L.H., Tytgat J., Sampaio S.V. (2019). First report on BaltCRP, a cysteine-rich secretory protein (CRISP) from *Bothrops alternatus* venom: Effects on potassium channels and inflammatory processes. Int. J. Biol. Macromol..

[26] Costa T.R., Menaldo D.L., Zoccal K.F., Burin S.M., Aissa A.F., Castro F.A.d., Faccioli L.H., Greggi Antunes L.M., Sampaio S.V. (2017). CR-LAAO, an L-amino acid oxidase from *Calloselasma rhodostoma* venom, as a potential tool for developing novel immunotherapeutic strategies against cancer. Sci. Rep..

[27] Li R., Tang Y., Chen Z., Liu Y. (2024). Screening TLR4 Binding Peptide from *Naja atra* Venom Glands Based on Phage Display. Toxins.

[28] Alquraini A., El Khoury J. (2020). Scavenger receptors. Curr. Biol..

[29] Gulati A., Kaur D., Krishna Prasad G., Mukhopadhaya A. (2019). PRR function of innate immune receptors in recognition of bacteria or bacterial ligands. Biochem. Biophys. Roles Cell Surf. Mol..

[30] Martínez V.G., Moestrup S.K., Holmskov U., Mollenhauer J., Lozano F. (2011). The conserved scavenger receptor cysteine-rich superfamily in therapy and diagnosis. Pharmacol. Rev..

[31] PrabhuDas M.R., Baldwin C.L., Bollyky P.L., Bowdish D.M., Drickamer K., Febbraio M., Herz J., Kobzik L., Krieger M., Loike J. (2017). A consensus definitive classification of scavenger receptors and their roles in health and disease. J. Immunol..

[32] Taban Q., Mumtaz P.T., Masoodi K.Z., Haq E., Ahmad S.M. (2022). Scavenger receptors in host defense: From functional aspects to mode of action. Cell Commun. Signal..

[33] Cho J.H., Sprent J. (2018). TCR tuning of T cell subsets. Immunol. Rev..

[34] Dong J., Zhang K., Hong J., Ye J., Wang L., Ke Q., Ye Y., Jin K., Chen Y., Wu J. (2025). The multiple functions of CD5 in diseases related to immune disorders. Ann. Med..

[35] Velasco-de Andrés M., Casadó-Llombart S., Català C., Leyton-Pereira A., Lozano F., Aranda F. (2020). Soluble CD5 and CD6: Lymphocytic class I scavenger receptors as immunotherapeutic agents. Cells.

[36] Vera J., Fenutría R., Cañadas O., Figueras M., Mota R., Sarrias M.-R., Williams D.L., Casals C., Yelamos J., Lozano F. (2009). The CD5 ectodomain interacts with conserved fungal cell wall components and protects from zymosan-induced septic shock-like syndrome. Proc. Natl. Acad. Sci. USA.

[37] Sarhan M.A., Pham T.N., Chen A.Y., Michalak T.I. (2012). Hepatitis C virus infection of human T lymphocytes is mediated by CD5. J. Virol..

[38] Sarrias M.-R., Farnós M., Mota R., Sánchez-Barbero F., Ibáñez A., Gimferrer I., Vera J., Fenutría R., Casals C., Yélamos J. (2007). CD6 binds to pathogen-associated molecular patterns and protects from LPS-induced septic shock. Proc. Natl. Acad. Sci. USA.

[39] Carrasco E., Escoda C., Alvarez-Fernández C., Sanchez-Palomino S., Carreras E., Gatell J.M., Gallart T., García F., Climent N., Lozano F. (2014). A role for scavenger-like lymphocyte receptor CD6 in HIV-1 viral infection. AIDS Res. Hum. Retroviruses.

[40] Miles S., Velasco-de-Andres M., Lozano F., Mourglia-Ettlin G. (2020). Interactome analysis of CD5 and CD6 ectodomains with tegumental antigens from the helminth parasite *Echinococcus granulosus sensu lato*. Int. J. Biol. Macromol..

[41] Mourglia-Ettlin G., Miles S., Velasco-De-Andres M., Armiger-Borras N., Cucher M., Dematteis S., Lozano F. (2018). The ectodomains of the lymphocyte scavenger receptors CD5 and CD6 interact with tegumental antigens from *Echinococcus granulosus sensu lato* and protect mice against secondary cystic echinococcosis. PLoS Neglected Trop. Dis..

[42] García-Luna J., Rivero-Osorio F., González-Porcile M.C., Arbildi P., Miles S., Magnone J., Velasco-De-Andrés M., Dematteis S., Lozano F., Mourglia-Ettlin G. (2024). Recombinant CD5 and CD6 Ectodomains Induce Antiparasitic and Immunomodulatory Effects in Secondary Cystic Echinococcosis. Parasite Immunol..

[43] Martínez-Florensa M., Consuegra-Fernández M., Martínez V.G., Cañadas O., Armiger-Borràs N., Bonet-Roselló L., Farrán A., Vila J., Casals C., Lozano F. (2014). Targeting of key pathogenic factors from gram-positive bacteria by the soluble ectodomain of the scavenger-like lymphocyte receptor CD6. J. Infect. Dis..

[44] Simarro M., Pelassy C., Calvo J., Places L., Aussel C., Lozano F. (1997). The cytoplasmic domain of CD5 mediates both TCR/CD3-dependent and-independent diacylglycerol production. J. Immunol..

[45] Wang Q., Du Q., Guo B., Mu D., Lu X., Ma Q., Guo Y., Fang L., Zhang B., Zhang G. (2020). A method to prevent SARS-CoV-2 IgM false positives in gold immunochromatography and enzyme-linked immunosorbent assays. J. Clin. Microbiol..

[46] Wang Q., Lei Y., Lu X., Wang G., Du Q., Guo X., Xing Y., Zhang G., Wang D. (2019). Urea-mediated dissociation alleviate the false-positive *Treponema pallidum*-specific antibodies detected by ELISA. PLoS ONE.

[47] Dauner J.G., Pan Y., Hildesheim A., Kemp T.J., Porras C., Pinto L.A. (2012). Development and application of a GuHCl-modified ELISA to measure the avidity of anti-HPV L1 VLP antibodies in vaccinated individuals. Mol. Cell. Probes.

[48] Laemmli U.K. (1970). Cleavage of structural proteins during the assembly of the head of bacteriophage T4. Nature.

[49] Arndt C., Koristka S., Feldmann A., Bachmann M. (2018). Native polyacrylamide gels. Electrophoretic Separation of Proteins: Methods and Protocols.

[50] Dutta S., Gogoi D., Mukherjee A.K. (2015). Anticoagulant mechanism and platelet deaggregation property of a non-cytotoxic, acidic phospholipase A2 purified from Indian cobra (*Naja naja*) venom: Inhibition of anticoagulant activity by low molecular weight heparin. Biochimie.

[51] Joubert F.J., Taljaard N. (1980). Purification, some properties and amino-acid sequences of two phospholipases A (CM-II and CM-III) from *Naja naja kaouthia* venom. Eur. J. Biochem..

[52] Stepanian L., Son I., Chalikian T.V. (2017). Effect of urea on protein-ligand association. Biophys. Chem..

[53] Pessenda G., Silva L.C., Campos L.B., Pacello E.M., Pucca M.B., Martinez E.Z., Barbosa J.E. (2016). Human scFv antibodies (Afribumabs) against Africanized bee venom: Advances in melittin recognition. Toxicon.

[54] Pucca M.B., Cerni F.A., Oliveira I.S., Jenkins T.P., Argemí L., Sørensen C.V., Ahmadi S., Barbosa J.E., Laustsen A.H. (2019). Bee updated: Current knowledge on bee venom and bee envenoming therapy. Front. Immunol..

[55] Wehbe R., Frangieh J., Rima M., El Obeid D., Sabatier J.-M., Fajloun Z. (2019). Bee venom: Overview of main compounds and bioactivities for therapeutic interests. Molecules.

[56] Doley R., Kini R. (2009). Protein complexes in snake venom. Cell. Mol. Life Sci..

[57] Laustsen A.H. (2016). Toxin synergism in snake venoms. Toxin Rev..

[58] McFarlane L.O., Pukala T.L. (2024). Proteomic investigation of cape cobra (*Naja nivea*) venom reveals first evidence of quaternary protein structures. Toxins.

[59] Mukherjee A.K. (2010). Non-covalent interaction of phospholipase A2 (PLA2) and kaouthiotoxin (KTX) from venom of *Naja kaouthia* exhibits marked synergism to potentiate their cytotoxicity on target cells. J. Venom Res..

[60] Wang C.R., Bubner E.R., Jovcevski B., Mittal P., Pukala T.L. (2020). Interrogating the higher order structures of snake venom proteins using an integrated mass spectrometric approach. J. Proteom..

[61] Xiong S., Huang C. (2018). Synergistic strategies of predominant toxins in snake venoms. Toxicol. Lett..

[62] Adamude F.A., Dingwoke E.J., Abubakar M.S., Ibrahim S., Mohamed G., Klein A., Sallau A.B. (2021). Proteomic analysis of three medically important Nigerian Naja (*Naja haje*, *Naja katiensis* and *Naja nigricollis*) snake venoms. Toxicon.

[63] Malih I., Tee T.Y., Saile R., Ghalim N., Othman I. (2014). Proteomic analysis of Moroccan cobra *Naja haje* legionis venom using tandem mass spectrometry. J. Proteom..

[64] Salama W.H., Shaheen M.N., Shahein Y.E. (2022). Egyptian cobra (*Naja haje haje*) venom phospholipase A2: A promising antiviral agent with potent virucidal activity against simian rotavirus and bovine coronavirus. Arch. Microbiol..

[65] Laraba-Djebari F., Legros C., Crest M., Ceard B., Romi R., Mansuelle P., Jacquet G., Van Rietschoten J., Gola M., Rochat H. (1994). The kaliotoxin family enlarged. Purification, characterization, and precursor nucleotide sequence of KTX2 from *Androctonus australis* venom. J. Biol. Chem..

[66] Bougis P.E., Rochat H., Smith L.A. (1989). Precursors of *Androctonus australis* scorpion neurotoxins: Structures of precursors, processing outcomes, and expression of a functional recombinant toxin II. J. Biol. Chem..

[67] Zerouti K., Khemili D., Laraba-Djebari F., Hammoudi-Triki D. (2021). Nontoxic fraction of scorpion venom reduces bacterial growth and inflammatory response in a mouse model of infection. Toxin Rev..

[68] Whiteley C.G. (2000). Enzyme kinetics: Partial and complete uncompetitive inhibition. Biochem. Educ..

[69] Kim H., Lee H., Lee G., Jang H., Kim S.-S., Yoon H., Kang G.-H., Hwang D.-S., Kim S.K., Chung H.-S. (2015). Phospholipase A2 inhibits cisplatin-induced acute kidney injury by modulating regulatory T cells by the CD206 mannose receptor. Kidney Int..

[70] Mukhopadhyay A., Stahl P. (1995). Bee venom Phospholipase A2Is recognized by the Macrophage Mannose receptor. Arch. Biochem. Biophys..

[71] Fontecilla-Camps J.C., Habersetzer-Rochat C., Rochat H. (1988). Orthorhombic crystals and three-dimensional structure of the potent toxin II from the scorpion *Androctonus australis hector*. Proc. Natl. Acad. Sci. USA.

[72] Khemili D., Valenzuela C., Laraba-Djebari F., Hammoudi-Triki D. (2019). Differential effect of *Androctonus australis hector* venom components on macrophage KV channels: Electrophysiological characterization. Eur. Biophys. J..

[73] Pucca M.B., Peigneur S., Cologna C.T., Cerni F.A., Zoccal K.F., de CF Bordon K., Faccioli L.H., Tytgat J., Arantes E.C. (2015). Electrophysiological characterization of the first *Tityus serrulatus* alpha-like toxin, Ts5: Evidence of a pro-inflammatory toxin on macrophages. Biochimie.

[74] Ramírez-Bello V., Sevcik C., Peigneur S., Tytgat J., D’Suze G. (2014). Macrophage alteration induced by inflammatory toxins isolated from *Tityus discrepans* scorpion venom. The role of Na^+^/Ca^2+^ exchangers. Toxicon.

[75] Mendes L.C., Viana G.M.M., Nencioni A.L.A., Pimenta D.C., Beraldo-Neto E. (2023). Scorpion peptides and ion channels: An insightful review of mechanisms and drug development. Toxins.

[76] Devaux C., Jouirou B., Krifi M.N., Clot-Faybesse O., El Ayeb M., Rochat H. (2004). Quantitative variability in the biodistribution and in toxinokinetic studies of the three main alpha toxins from the *Androctonus australis hector* scorpion venom. Toxicon.

[77] Vogel C.-W., Bredehorst R., Fritzinger D.C., Grunwald T., Ziegelmüller P., Kock M.A. (1996). Structure and function of cobra venom factor, the complement-activating protein in cobra venom. Natural Toxins 2: Structure, Mechanism of Action, and Detection.

[78] Vogel C.-W., Fritzinger D.C. (2010). Cobra venom factor: Structure, function, and humanization for therapeutic complement depletion. Toxicon.

[79] Gutiérrez J.M., Lomonte B. (2013). Phospholipases A2: Unveiling the secrets of a functionally versatile group of snake venom toxins. Toxicon.

[80] Oliveira A.L., Viegas M.F., da Silva S.L., Soares A.M., Ramos M.J., Fernandes P.A. (2022). The chemistry of snake venom and its medicinal potential. Nat. Rev. Chem..

[81] Al-Quraishy S., Dkhil M.A., Abdel Moneim A.E. (2014). Hepatotoxicity and oxidative stress induced by *Naja haje* crude venom. J. Venom. Anim. Toxins Incl. Trop. Dis..

[82] Mejri H., Mokrani R., Ksouri A., Seddik M., Awad N., Ayme G., Chagour T., Mokrani A., Louchene C.E., Salhi I. (2024). Neutralizing nanobodies against venoms from *Naja haje* species captured in north africa. Toxins.

[83] Silva A., Hodgson W.C., Isbister G.K. (2017). Antivenom for neuromuscular paralysis resulting from snake envenoming. Toxins.

[84] Bickler P.E. (2020). Amplification of snake venom toxicity by endogenous signaling pathways. Toxins.

[85] Ivanušec A., Šribar J., Veranič P., Križaj I. (2022). The phospholipase activity of ammodytoxin, a prototype snake venom β-neurotoxin, is not obligatory for cell Internalisation and translocation to mitochondria. Toxins.

[86] Cedro R.C., Menaldo D.L., Costa T.R., Zoccal K.F., Sartim M.A., Santos-Filho N.A., Faccioli L.H., Sampaio S.V. (2018). Cytotoxic and inflammatory potential of a phospholipase A 2 from *Bothrops jararaca* snake venom. J. Venom. Anim. Toxins Incl. Trop. Dis..

[87] Deka A., Sharma M., Sharma M., Mukhopadhyay R., Doley R. (2017). Purification and partial characterization of an anticoagulant PLA2 from the venom of Indian *Daboia russelii* that induces inflammation through upregulation of proinflammatory mediators. J. Biochem. Mol. Toxicol..

[88] Echeverria S., Leiguez E., Guijas C., do Nascimento N.G., Acosta O., Teixeira C., Leiva L.C., Rodríguez J.P. (2018). Evaluation of pro-inflammatory events induced by *Bothrops alternatus* snake venom. Chem.-Biol. Interact..

[89] Moreira V., Gutiérrez J.M., Soares A.M., Zamunér S.R., Purgatto E., Teixeira C.d.F.P. (2008). Secretory phospholipases A2 isolated from *Bothrops asper* and from *Crotalus durissus terrificus* snake venoms induce distinct mechanisms for biosynthesis of prostaglandins E2 and D2 and expression of cyclooxygenases. Toxicon.

[90] Fry B.G., Roelants K., Champagne D.E., Scheib H., Tyndall J.D., King G.F., Nevalainen T.J., Norman J.A., Lewis R.J., Norton R.S. (2009). The toxicogenomic multiverse: Convergent recruitment of proteins into animal venoms. Annu. Rev. Genom. Hum. Genet..

[91] Elieh Ali Komi D., Shafaghat F., Zwiener R.D. (2018). Immunology of bee venom. Clin. Rev. Allergy Immunol..

[92] Calixto M., Triches K., Calixto J. (2003). Analysis of the inflammatory response in the rat paw caused by the venom of *Apis melifera* bee. Inflamm. Res..

[93] Hartman D., Tomchek L., Lugay J., Lewin A., Chau T., Carlson R. (1991). Comparison of antiinflammatory and antiallergic drugs in the melittin-and D49 PLA2-induced mouse paw edema models. Agents Actions.

[94] Palm N.W., Rosenstein R.K., Yu S., Schenten D.D., Florsheim E., Medzhitov R. (2013). Bee venom phospholipase A2 induces a primary type 2 response that is dependent on the receptor ST2 and confers protective immunity. Immunity.

