# Supplementary figures and images for: Interaction of Human Lymphocyte Scavenger Receptors CD5 and CD6 with Toxins from Naja haje, Androctonus australis and Apis mellifera Venoms
Source: Biomolecules. 2026 May 5;16(5):681. doi: 10.3390/biom16050681 (PMC13204495; doi:10.3390/biom16050681)

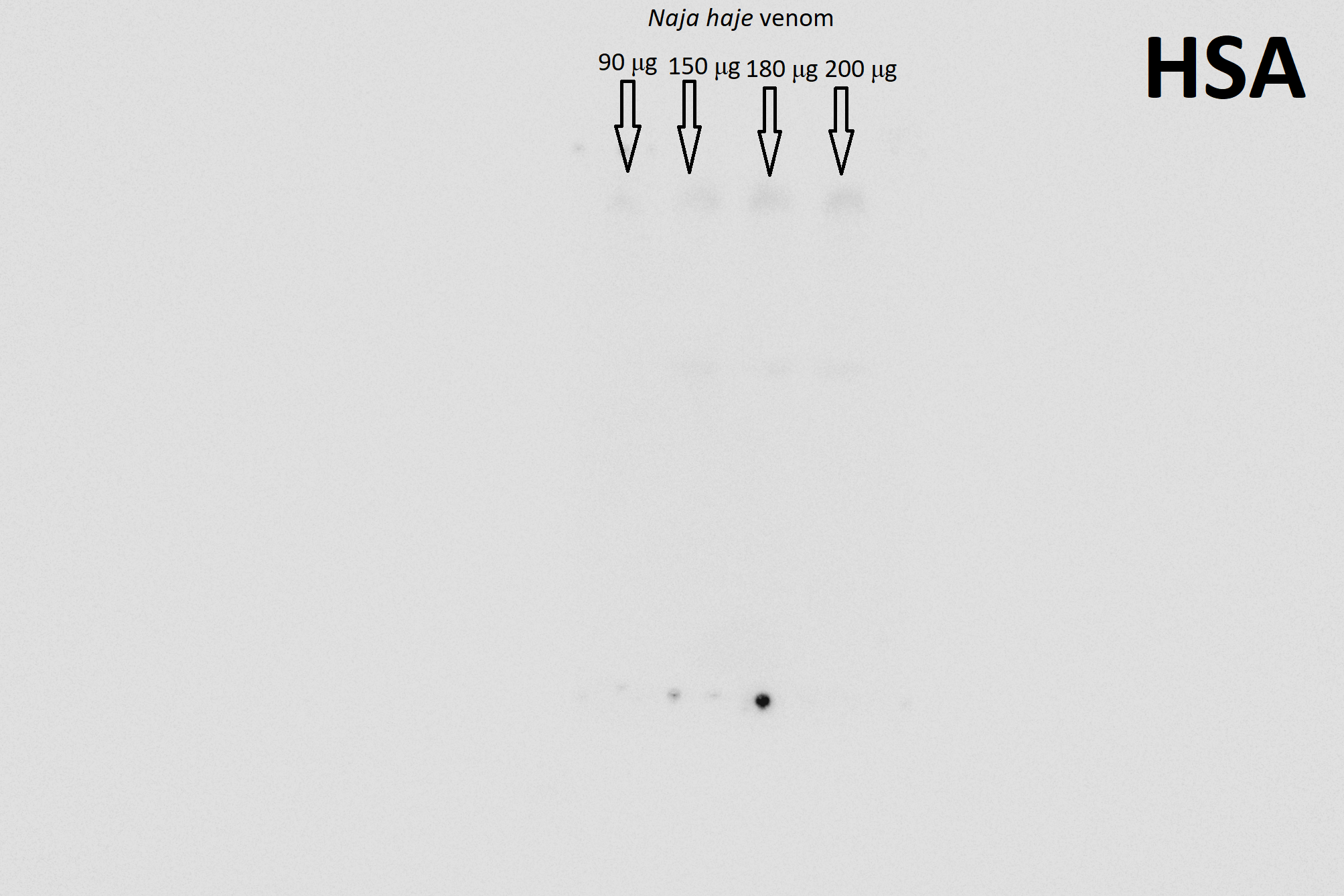

Supplement: Supplementary file 1 [file biomolecules-16-00681-s001.zip › Original image for Figure 7A-HSA.png]

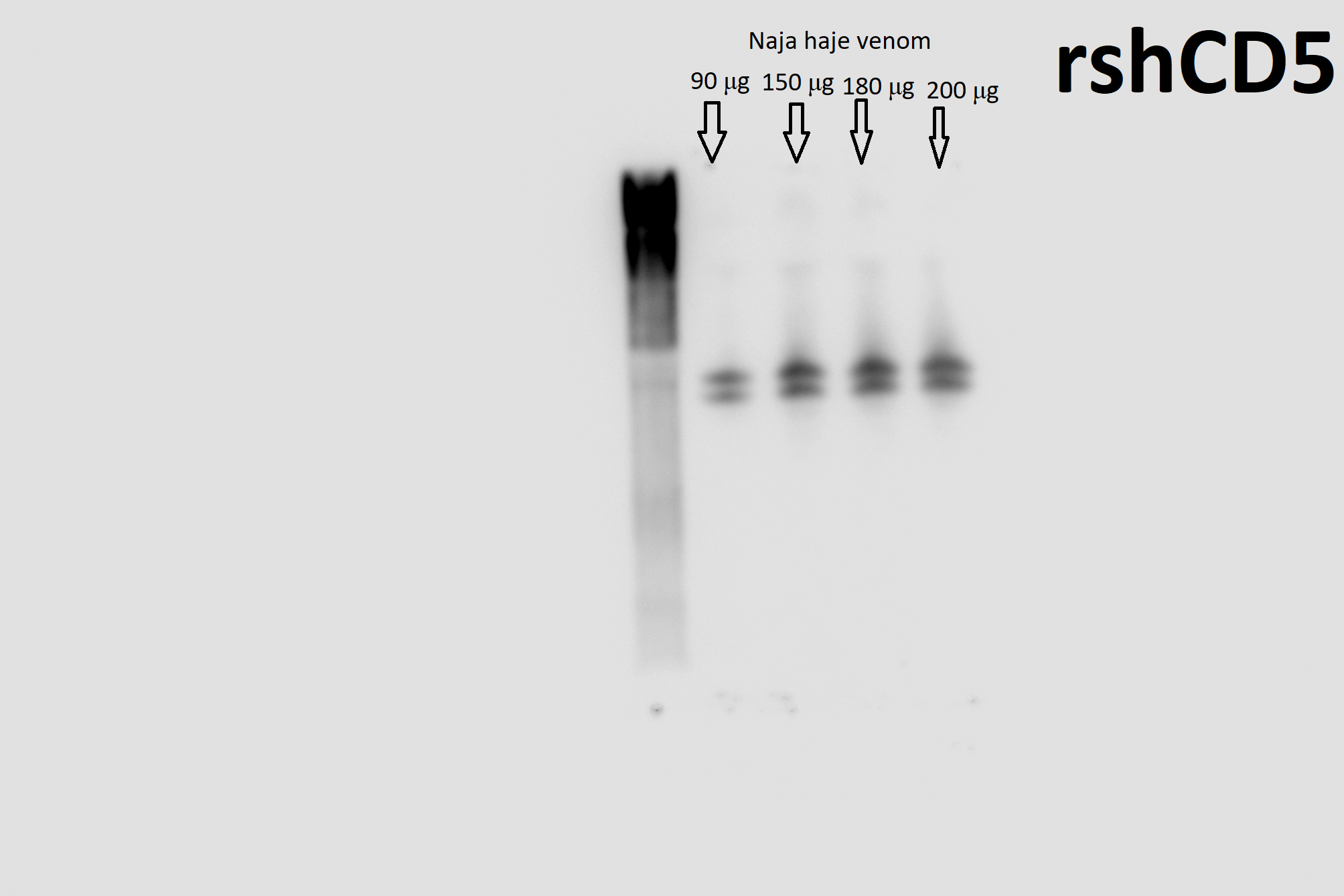

Supplement: Supplementary file 1 [file biomolecules-16-00681-s001.zip › Original image for Figure 7A-rshCD5.png]

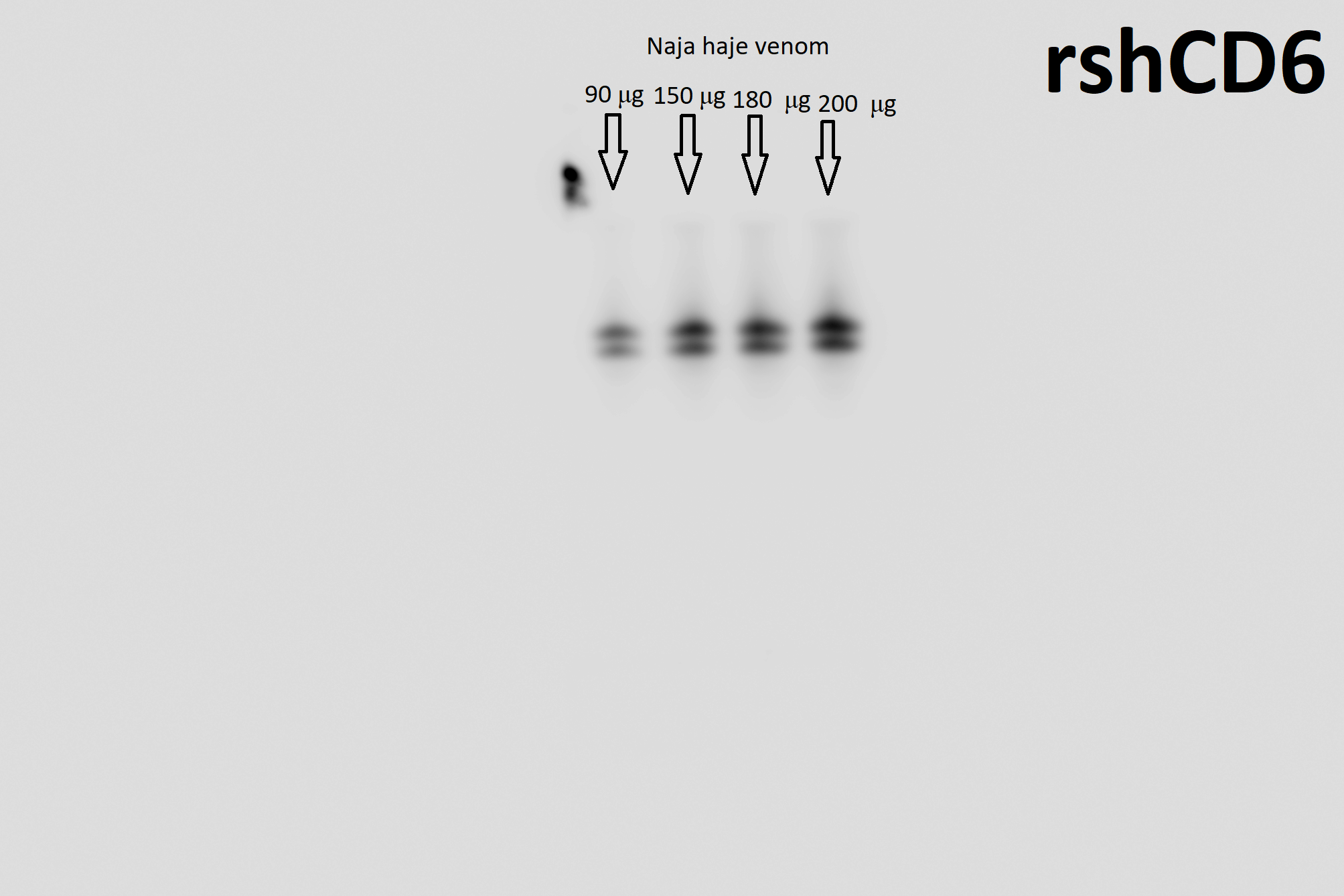

Supplement: Supplementary file 1 [file biomolecules-16-00681-s001.zip › Original image for Figure 7A-rshCD6.png]

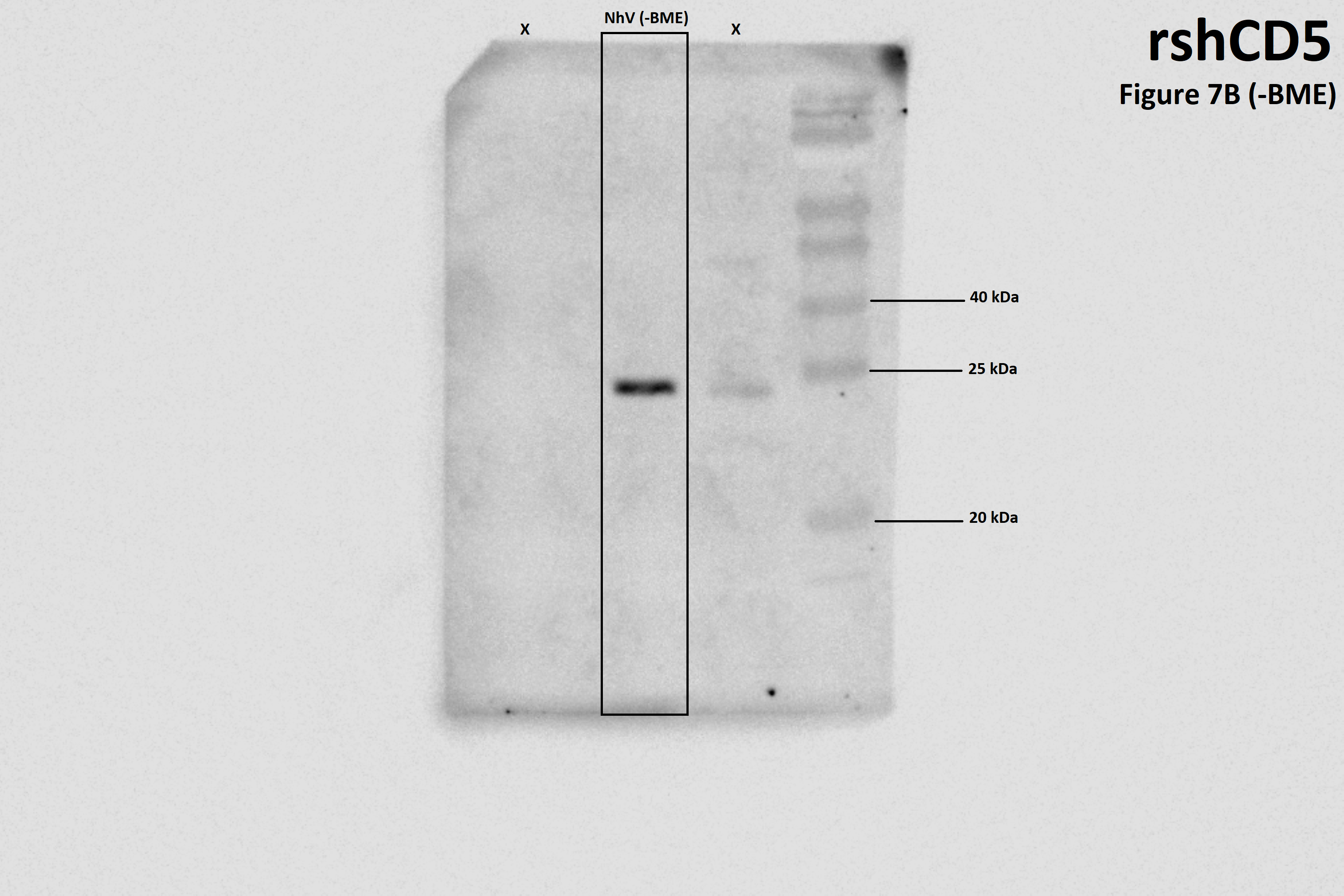

Supplement: Supplementary file 1 [file biomolecules-16-00681-s001.zip › Original image for Figure 7B-rshCD5(-BME) .png]

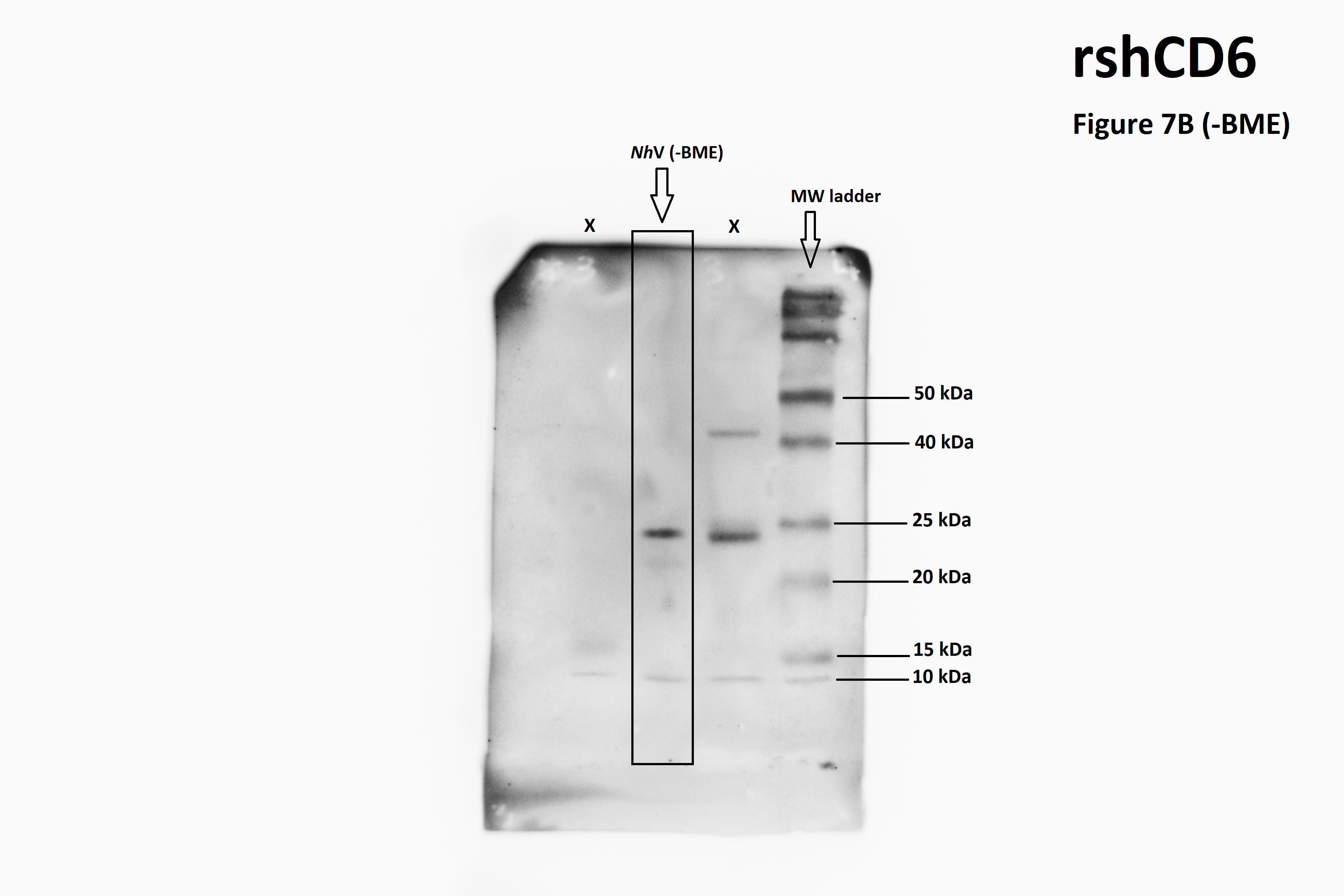

Supplement: Supplementary file 1 [file biomolecules-16-00681-s001.zip › Original image for Figure 7B-rshCD6(-BME) .png]

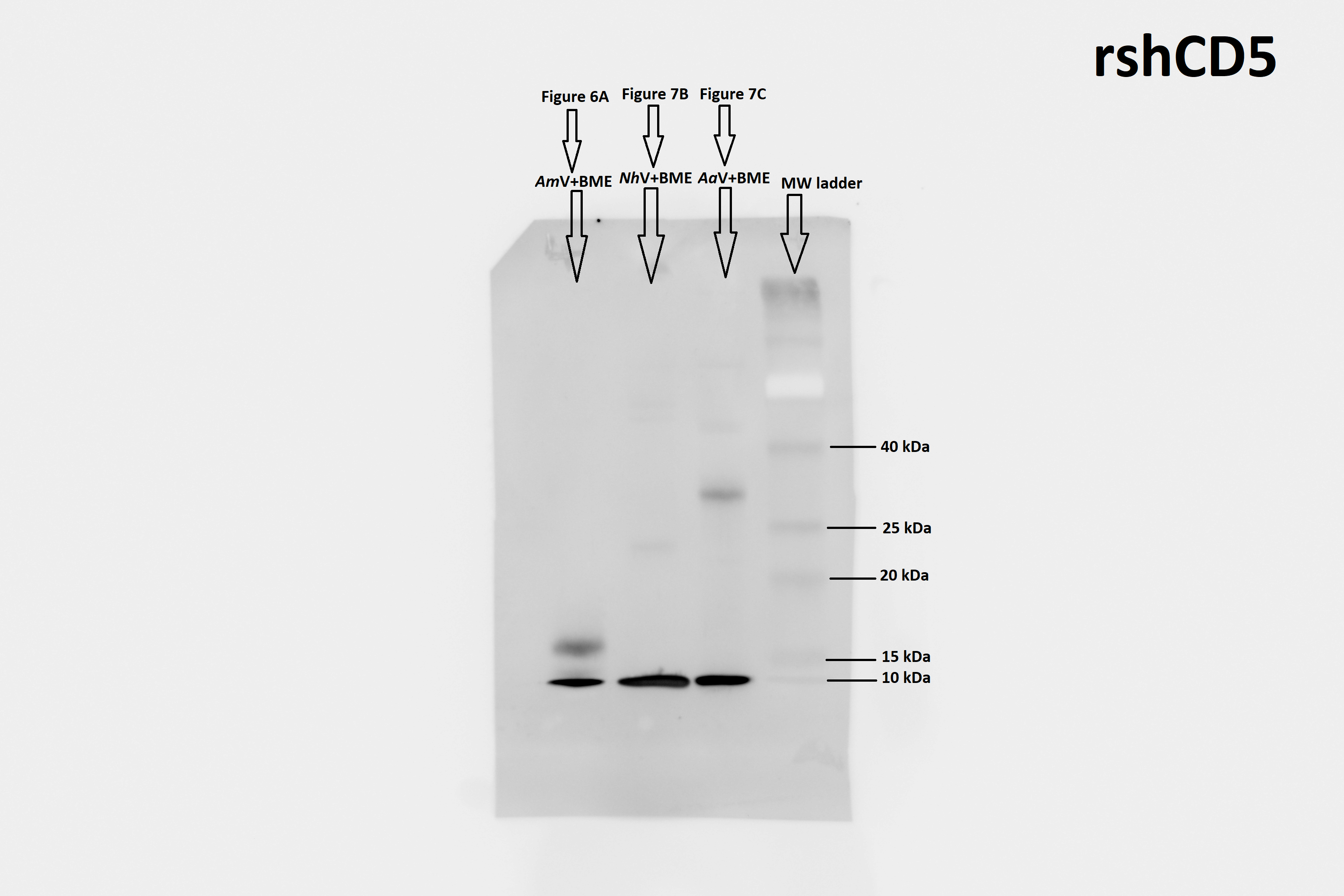

Supplement: Supplementary file 1 [file biomolecules-16-00681-s001.zip › Original image for Figures 6A, 7B and 7C-rshCD5(+BME).png]

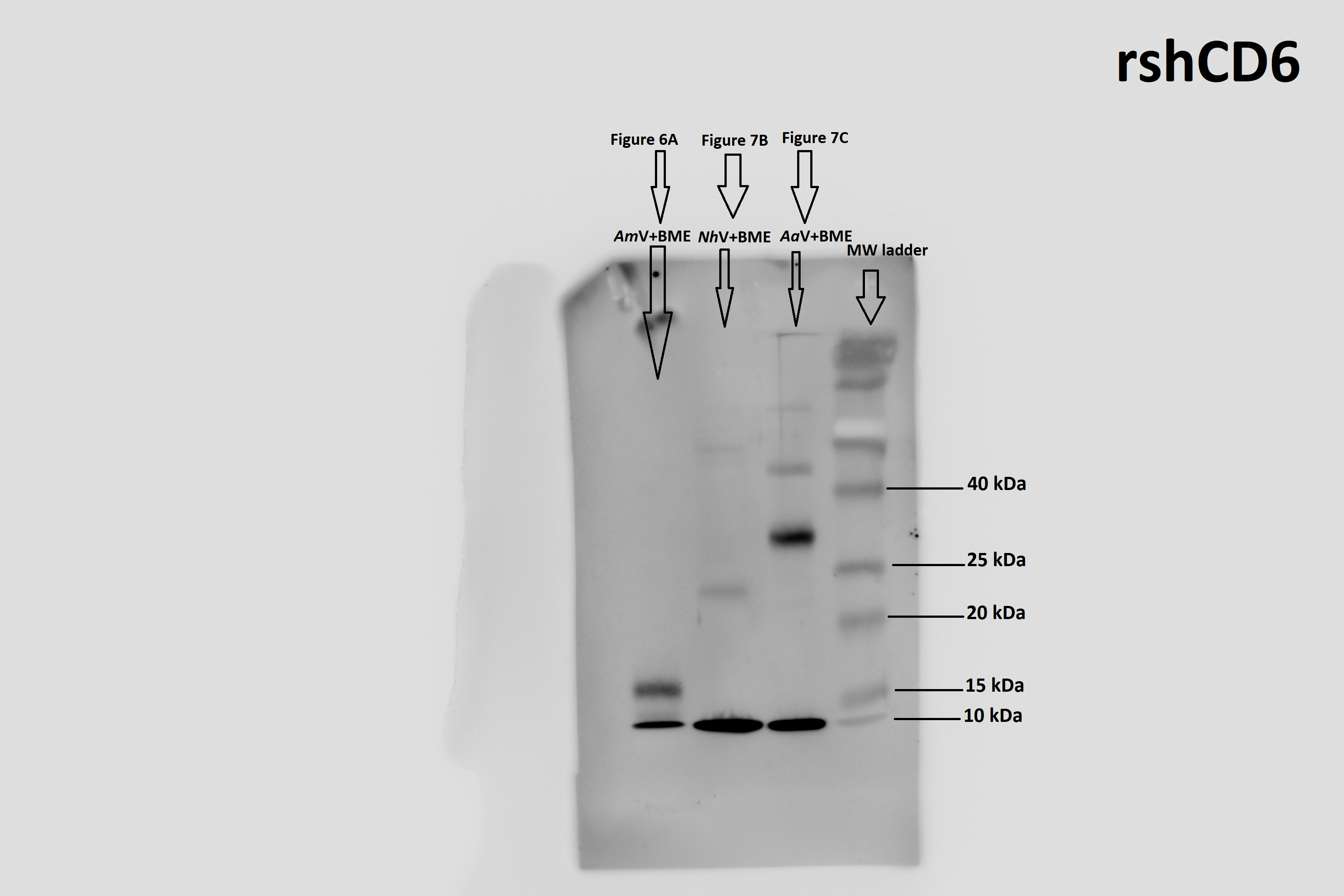

Supplement: Supplementary file 1 [file biomolecules-16-00681-s001.zip › Original image for Figures 6A, 7B and 7C-rshCD6(+BME).png]
